# Using leap motion to investigate the emergence of structure in speech and language

**DOI:** 10.3758/s13428-016-0818-x

**Published:** 2016-11-08

**Authors:** Kerem Eryilmaz, Hannah Little

**Affiliations:** 1 Vrije Universiteit Brussel, Artificial Intelligence Laboratory, Pleinlaan 2, 1050 Brussels, Belgium; 2 Max Planck Institute for Psycholinguistics, Language and Cognition Department, Wundtlaan 1, 6525 XD, Nijmegen, The Netherlands

**Keywords:** Artificial language learning, Language evolution, Leap Motion, Python, Signal space proxies, Combinatorialstructure

## Abstract

In evolutionary linguistics, experiments using artificial signal spaces are being used to investigate the emergenceof speech structure. These signal spaces need to be continuous, non-discretized spaces from which discrete unitsand patterns can emerge. They need to be dissimilar from—but comparable with—the vocal tract, in order tominimize interference from pre-existing linguistic knowledge, while informing us about language. This is a hardbalance to strike. This article outlines a new approach that uses the Leap Motion, an infrared controller that canconvert manual movement in 3d space into sound. The signal space using this approach is more flexible than signalspaces in previous attempts. Further, output data using this approach is simpler to arrange and analyze. Theexperimental interface was built using free, and mostly open- source libraries in Python. We provide our sourcecode for other researchers as open source.

## Introduction

In evolutionary linguistics, artificial language learning (ALL) experiments are becoming increasingly commonplace (Scott-Phillips and Kirby 2010). How humans in the laboratory learn, use and transmit artificial languages can inform our knowledge of how linguistic structure came about via transmission and communication. Previously, these experiments have focused on the emergence of structure on a morphosyntactic level using artificial mini-languages composed from small discrete building blocks (e.g., Kirby *et al*. 2008). However, there is a growing body of work that is using artificial signaling paradigms to investigate the emergence of combinatorial structure; the level of structure where meaningless building blocks combine to make morphemes or words. Within this work, it does not make sense to initially construct artificial signals from discrete building blocks, as it is the emergence of discrete building blocks, which is of interest. Accordingly, this work uses continuous signal spaces to investigate how combinatorial building blocks emerge in linguistic signals.

In this paper, we will briefly review existing continuous paradigms before presenting the functionality of our own paradigm, which uses the Leap Motion sensor to produce auditory feedback. Further, we present explicit instructions for implementation as an appendix. You can find the download instructions in “Getting the framework”.

## Artificial signal spaces

The ideal artificial signal space to investigate the emergence of combinatorial categories and structure is one that prevents interference from pre-existing linguistic knowledge, whilst having a continuous space from which discrete elements can emerge. Here, we will briefly outline issues with continuous signal spaces that have been used in artificial language experiments to investigate the emergence of combinatorial structure. Specifically, we will discuss the problem of interference from iconicity, the problem of data, which is difficult or labor-intensive to analyze, and the problem of having restrictions on the shape and size of artificial signal spaces. This breakdown will hopefully help illustrate how our own paradigm improves on previous work.

### Limiting opportunity for iconicity

The use of graphical signals to investigate trends in how communication systems emerge and evolve started with the use of graphical symbols by Healey et al. (2002), and has since grown into its own field of research: “experimental semiotics” (for a review see Galantucci and Garrod, 2011; Galantucci et al., 2012). Experimental Semiotics has significant overlap with ALL experiments in language evolution and is not confined to only graphical signals. However, many of these experiments investigate the effects of communication and transmission by using graphical pictionary-style communication tasks where participants are given a concept to communicate without the use of words (e.g., Garrod et al., 2007; Fay et al., 2008). These experiments are useful for investigating processes such as conventionalization. However, experiments investigating the emergence of combinatorial structure become difficult to design with graphical paradigms, as participants are very familiar with presenting content graphically, both using written language and creating iconic representations via drawing. In addition to this, graphical interfaces make it easy to utilize iconicity, which has been shown to affect the emergence of combinatorial structure (see for instance Roberts et al., 2015 and Verhoef et al., 2015a). Further, different levels of iconicity are possible using different linguistic modalities (Fay et al. 2014), meaning that trends demonstrated with paradigms offering a lot of available iconicity (drawing) may not be extrapolatable to communication mediums with less available iconicity (e.g., speech).

In order to combat these issues of iconicity, Galantucci (2005) developed an approach that used a graphical interface, but had constraints on what participants could do using the apparatus. The interface is a stylus that writes on virtual paper that has a constant downwards drift, so participants can only control signals on the horizontal dimension. Using this approach, Galantucci has conducted social coordination experiments to investigate how communication systems with combinatorial structure can emerge (Galantucci 2005; Roberts and Galantucci 2012), and more specific experiments have been done, which have looked at how rapidity of fading (how quickly signals disappear after production) affects combinatorial structure in signals (Galantucci et al. 2010), and how the potential for iconicity affects the emergence of combinatorial structure (Roberts et al. 2015). There have also been experiments that have used the apparatus in an iterated learning paradigm, where participants’ outputs were fed to other participants in transmission chains to investigate whether combinatorial structure emerges more reliably via vertical transmission (learning) in contrast with horizontal transmission (communication) (Del Giudice 2012).

### Ease of analysis

Verhoef has done several experiments that use slide whistles as a proxy for an articulation space (e.g., Verhoef et al., 2014). Her experiments involve participants learning pre-recorded whistles and reproducing them from memory. This has been implemented in an iterated learning experiment to see if learning biases within transmission chains could influence the emergence of combinatorial structure within an inventory of whistles. Verhoef has implemented this in conditions with meanings to investigate the role of iconicity (Verhoef et al. 2015a) and without meanings, to isolate only the role of learning biases (Verhoef et al. 2014). Results have clearly shown that within transmission chains, combinatorial structure emerges and signals become more learnable. However, the output from these experiments is audio recordings of the acoustic signals, meaning that quite a bit of processing is required in order to extract the relevant data (the pitch values at each time frame) before analysis can start. Further, the relationship between stopper-movement and signal-pitch is not linear, and manipulating the mapping between stopper and pitch is not possible. This not only restricts possible experimental designs, but also complicates analysis if the experiment wishes to calculate something like the stopper position from the pitch data.

Since these initial experiments, a computational alternative to the slide whistle has been developed, which can work with a mouse on a screen, or via touch pads on tablets. In one experiment, participants created signals by placing their finger on a virtual slide whistle app on a tablet (Verhoef et al. 2015b). This experiment explored whether some meanings being more easily mappable than others would facilitate communicative success. This has undoubtedly solved the problem of having data that can be analyzed quickly without much processing^1^ Verhoef has since used a digital signaling apparatus but without auditory feedback, simply having visual signals represented by a bubble that can be moved up and down using a touchscreen (Verhoef et al. 2016).

Both (Galantucci 2005) and Verhoef’s slide whistle experiments have used gaps in the signals to measure structure. The analysis (e.g., in Roberts et al., 2015) starts with a signal already segmented into “forms” where signal parts are separated by a gap (e.g., the stylus lifting off, or the participant stops blowing into the whistle). In other words, the participants were allowed to leave marks at segment boundaries. These boundaries are problematic because they are pre-conventionalized markers for structure. Instead of relying on statistical regularities or negotiating a boundary marker themselves, they are given an explicit way to segment the signals. Comparable cues are available in written language (such as spaces between words), but not necessarily in human speech, and certainly not at the phonemic level.

### Flexibility of the signal space

Using slide whistles, the signal space is difficult to manipulate in its shape. An attempt has been made to affect the size of the signal space using slide whistles by putting a stopper on the plunger (Little and De Boer 2014). Reducing the range of pitches that could be produced using the whistle was hypothesized to make combinatorial structure emerge more quickly. Using the stylus paradigm, it is also difficult to manipulate the size, shape or dynamics of the signal space because it is confined to one dimension, causing researchers to manipulate the meaning space, rather than the signal space, to investigate the effect of things such as mappability (e.g., in Roberts et al., 2015).

Using digital slide whistles, it is easier to manipulate the shape of a signal space, and an experiment has been done that looks at the effects of different biases created by nonlinear mappings between the signal-space and the auditory feedback (Janssen et al. 2016). However, being on a flat surface, participants are still often tempted to produce signals as if they were graphical, focusing on the articulation space, rather than the auditory aspects of the signal. Because of this, the digital paradigms mentioned here have so far been limited to a 1 dimensional space (usually pitch). With a 2 dimensional space on a flat surface, participants are even more tempted to just “draw” their referent, as happened in de Boer and Verhoef (2012).

## The Leap Motion framework

The Leap Motion framework uses a commercially available, inexpensive, USB-powered sub-millimeter precision infrared hand-tracking device called Leap Motion (Holz 2014). It is a small rectangular box that sits on a desk with an upward-facing camera. It works by building a skeletal hand model from the infrared images it takes, converting each image to a data frame representing the hand(s) it was able to detect in that image. It is able to keep track of individual hands and the associated fingers, as well as their positions, orientations, and velocities.

The proxy we have developed using Leap Motion is conceptually similar to the musical instrument, the theremin. As the participant moves one hand above the sensor, the position of their hand is translated into an auditory tone. In our experiments, participants were only allowed to use one hand and we tracked their palm location, but there is nothing in the framework to limit the number of hands or tracking of specific fingers. The framework is flexible in what features of each Leap frame (or group of frames) would modulate aspects of an auditory signal. Experiments (or “Extherements”) are not necessarily limited to using the position of hands or fingers, but could also use, for example, the angle of the palm with the horizontal plane, or any arbitrary function of one or more data frames.

### Advantages of the Leap Motion approach

We have developed a signal space proxy, which we feel improves on the issues raised with previous methods above. Namely, opportunities for iconicity can be controlled, it improves the ease of analysis and, most importantly, is flexible in its geometry, size and in the nature of the signals it can produce. Further, the framework itself is flexible, allowing for use in many different experimental paradigms. Below is a list of all of the crucial features that the framework has, making it fit for purpose.

#### Continuous signal space

As already mentioned, a continuous signal space is needed to allow for the discretization of signal building-blocks and the emergence of combinatorial structure.

#### Minimizes interference from pre-existing linguistic knowledge

The Leap Motion framework converts hand movement into auditory feedback. While it is true that gesture is used in communication almost ubiquitously, the gesture that produces signals using this framework is not similar. For one, precise placement of the palm of one hand is not an important feature of co-speech gesture or gesture in sign language. Further, the use of hand-placement to generate precise auditory feedback is not something that occurs in natural language. However, both visual and acoustic signaling may help contribute to the ecological validity of experiments using the framework.

#### Limits opportunity for iconicity

The Leap Motion framework generates auditory signals that are less iconic than graphic signals. The framework still has a visual element (i.e., the hand position in front of the participant) and, as a result, participants still use this information to try and generate iconic signals. However, it is the auditory signals that are transmitted, not the visual ones, which makes iconicity a less salient feature in the transmitted signals. Iconicity in signals could be combated by transforming mappings between hand-position and auditory feedback, or by designing the signal space to be less intuitive with a given meaning space. Importantly, the framework offers flexibility to make the opportunity for iconicity more or less possible.

#### Ease of analysis

The signals in our framework start as raw, numeric data that can be converted into perceivable signals such as sounds, eliminating a whole step in the data analysis pipeline. This also makes it possible to automate analyses once the experimental session is over. The capabilities of data analysis using the Leap Motion framework are covered more extensively in “Data Output”.

#### Flexible signal space

It is possible to change the shape and dimensionality of the signal space using the Leap Motion framework. Having a flexible signal space makes it easier to make a signal space more or less like a signal modality used in natural languages. For example, slide whistles are more constrained than speech, but the results from these experiments are extrapolated to be relevant to speech. The current framework allows for the signal space to be made to be more or less like speech, or more or less like gesture, in order to answer whether previous signal spaces have ecological validity. Further, it allows for comparison of data from signal spaces that differ in a feature, perhaps relevant to the differences between the spoken and manual modalities or differences between previously used signal space proxies.

#### Ease of deployment

Experimental tools should be easy to deploy. Most researchers are not software developers, and thus prefer relatively simple, pre-configured frameworks that works out of the box. The Leap Motion framework comes with a default configuration that runs a peer-to-peer experiment for two participants, and it pre-packages all dependencies it legally can. It is designed for the use of non-programmers, and configuration changes are made by editing plain text files.

#### Flexible experimental structure

Our framework not only allows replicating the experiments that we have done utilizing it, but also allows easy implementation of new peer-to-peer artificial language learning tasks by extending the framework. Care has been taken to keep the implementation as modular and configurable as possible, so that the structure and the flow of the experiment (e.g., how interacting agents are chosen, in what order they interact, what are the phases etc.) can be altered with minimal impact to the rest of the functionality.

#### Open access and extensibility

A critical property, which functions as a precondition to some of the properties above, is the codebase being open access. This not only enables extensibility of the framework by third parties without asking the original developers, but also frees the user base from having to rely on the original developers for bug fixes. Our framework is open source and is free to use and modify.

### Getting the framework

The framework has been developed using a Python-based library to serve as an experimental workbench. The framework requires an ordinary modern desktop or laptop computer to run the experiments. As many computers as there are clients are required for practical reasons, and another computer to run the server, if the experimenter would like to keep track of the experiment throughout on a separate machine, though there’s no reason the server cannot be run on the same machine as a client. The framework does not have any specific hardware requirements apart from a Leap Motion sensor.

The code is available at https://github.com/keryil/leaparticulatorqt. It is open source, and the readers can use, modify, and distribute it as they wish, as well as contact the authors with questions or suggestions. The GitHub page also links to a quick start implementation video on YouTube.

### The signals

**Fig. 1:**
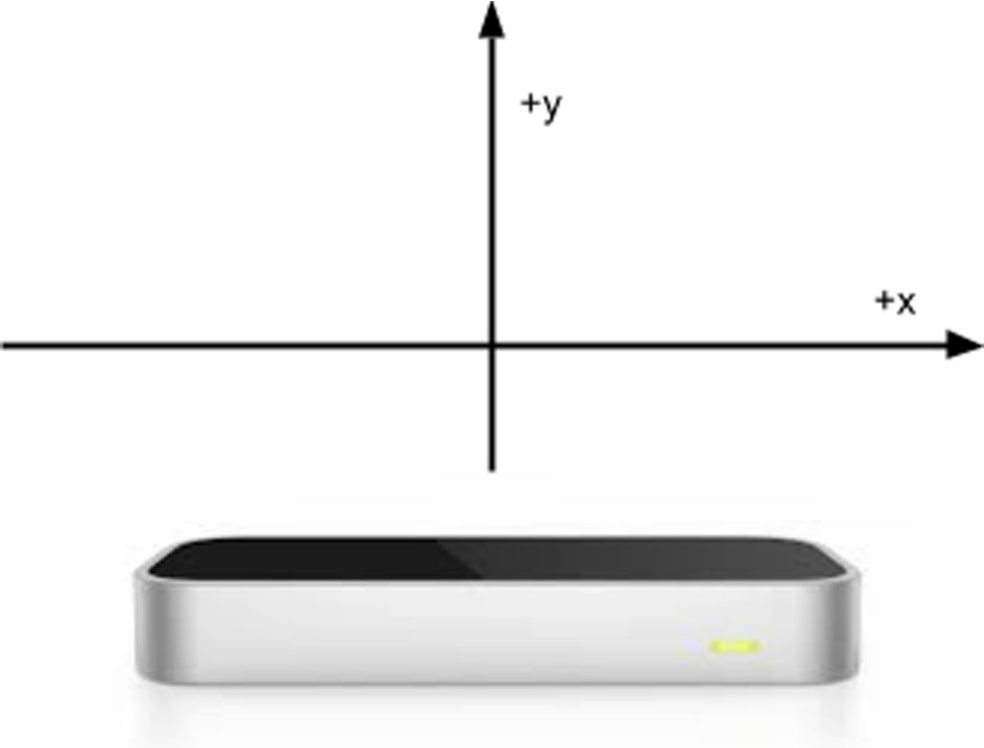
The Leap Motion controller showing the spatial dimensions used in one of our experiments. There is also the possibility to use the front-back dimension

Auditory signals are produced by moving one hand above the Leap Motion. The signals can be manipulated along any of the dimensions or features that the Leap Motion can detect. The paradigm records hand position above the Leap in three spatial dimensions and the duration of the signal can also be measured or controlled. Different auditory dimensions can be paired with the different spatial dimensions above the Leap Motion (see Fig. 1). For example if the experimenter wishes to have signals be manipulated on the dimension of pitch, then the pitch of signals can be affected by moving the hand left and right, back and front or up and down. Not every dimension needs to be paired with an auditory dimension, or even to auditory output at all (if one wanted to do a purely gestural or visual experiment for instance).

The mapping between hand position and feedback can be linear, but does not need to be. Signals can be manipulated by pitch (e.g., low to high from left to right) or volume (e.g., quiet to loud from up to down). In our experiments, we have not used mappings that were linear, because in pilot experiments participants found it much more difficult to perceive signal differences at the quiet and high ends of the signal space when the mappings were linear (the transformations used are available in Appendix “A.4 Signals”). It is also possible to produce the signal based on a non-linear combination of multiple dimensions or features, as long as they can be calculated from the Leap frames.

In the current framework, there is a strategy whereby when a hand is withdrawn from the signal space during an experiment, the amplitude of the signal gradually diminishes to zero within less than half a second, instead of cutting it off right away. This dramatically reduces auditory artifacts such as clicks, and makes the resulting signal sound more continuous-sounding.

**Fig. 2:**
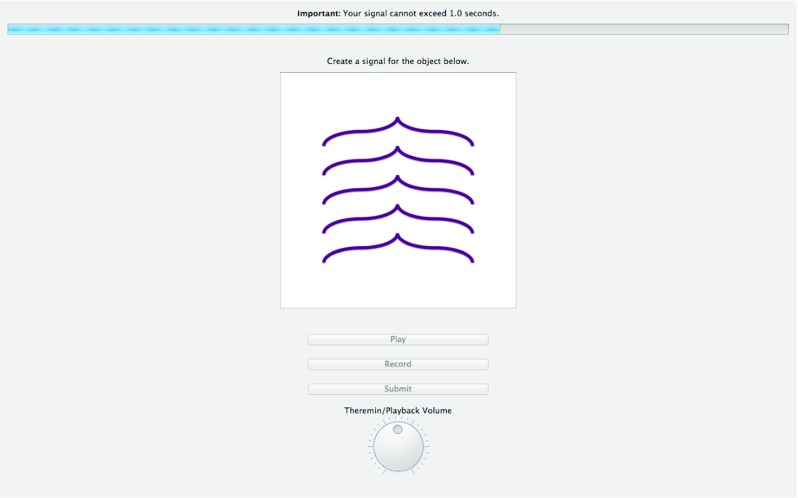
Signal creation screen for an experiment with limited signal durations. The *progress bar* indicates the time left until maximum duration is reached, though not all experiments will have a time limit on signals

There is functionality to have duration constraints on the signals in experiment, and to have a progress bar show participants time elapsing if there is a time constraint on signals (see Fig. 2, also “A.4.2 Manipulation of duration” in the Appendix).

### The Meanings

Usually, in artificial signaling experiments, signals refer to meanings (though this is not always the case, see Verhoef et al., 2014). Within the current framework, any image can serve as a meaning. As long as the meaning files follow a naming convention that follows their features, it is easy to select a subset of the meanings based on specified features.

### Experimental paradigms

Artificial language learning experiments generally come in three flavors: 1. individual learning or signal creation experiments, where individual participants create or learn and reproduce signals (e.g., Little et al., 2015; Little et al., 2016b), 2. iterated learning experiments, where the reproductions of one individual are taught to the next participant in a transmission chain (e.g., Kirby et al., 2008; Verhoef et al., 2014), and 3. communication games (e.g., Garrod et al., 2010; Roberts et al., 2015), where two or more participants use a created language to communicate with one another about a set of meanings. Several studies have even included a combination of these, e.g., having generations of communication games (e.g., Kirby et al., 2015; Verhoef et al., 2015b).

From a practical, interface-building perspective, nearly all these paradigms can be characterized as being made up, either partially or wholly, from components of peer-to-peer communication games where peers try to invent, learn or transmit signals through pairwise interactions with one another. All such games typically require at least one participant that produces signals and one that recognizes them, but it is perfectly possible for a single participant to assume both roles. For instance, individual learning tasks are communication games where a single participant acts both as the speaker and the hearer, effectively functioning as their own partner or peer in a pairwise interaction. They produce signals, and later, they are asked to recognize their own signals. Iterated learning tasks can also be described as identical-peer communication games, but with the difference that the peer (who once again is both the hearer and the speaker) is changed several times during the experiment to model vertical transmission. Therefore, throughout the current paper we shall review the communication game as the most general paradigm. However, all instantiations of ALLs are possible with the current framework.

Several experiments have already used the Leap Motion framework. These have mostly been individual signal creation experiments, for example (Little et al. 2015), which looked at the differences in signal structure between signals for meanings that differed along continuous dimensions compared to discrete differences, and (Little et al. 2016a), which looked at the effects of different signal dimensionalities on signal structure. There are also upcoming publications on a communication game (some details given in this manuscript), comparing structure and iconicity in signals created in communication or an individual signal creation task.

### Structure of experiments

Different experiments need to be structured in different ways, but for the most part, individual learning, iterated learning and communication experiments have a finite number of possible parts to the experiment. They usually need a window to create or reproduce signals, one to recognize signals, and one to provide feedback. The creation/reproduction tasks may be presented in batches or interleaved with the recognition tasks. In the current implementation, both are possible.

Experiments within the framework operate by exchanging message objects back and forth between a server and the client(s). Both the server and client are limited to sending and receiving a single line at a time, where that single line is a serialized form of the message object. There are mechanisms within the framework to ensure that the system fails when it receives unexpected, out-of-order messages to ensure the experiment is flowing exactly the way it should. The framework allows the experimenter to extend the functionality and flexibility of experiment designs by implementing new message classes without interfering with the rest of the messaging scheme.

#### Phases

Within the framework, the experiment design may be aided by the use of phases (blocks of tasks within an experiment that may be repeated). Between phases, different meaning spaces and different signal spaces can be used. For example, the meaning space might need to grow between phases after a set number of meanings have been seen, or a set number of interactions have happened. In our communication game, whether the meaning space grew was dependent on how successful participants had been at communicating the meanings they had seen up until that point. The idea being, that if they hadn’t established signals for existing meanings, they were unlikely to deal with new meanings well.

Each session can keep track of how successful participants are at communicating each meaning. We call “established meanings” any meaning that has been successfully communicated at least twice in a row. All other meanings, as well as established meanings that have recently been communicated incorrectly, are not “established”. By default, the meaning space expands if and only if all the current meanings are “established”, but the experimenter can add any criteria for progression to a new phase of the experiment. It is possible to add arbitrary logic that changes the signal space (e.g., add dimensions, swap dimensions, expand, shrink, etc.).

The framework also allows the probability of choosing a particular meaning as the topic at a particular round to be dependent on whether or not a meaning is established. At each communication round, there is a probability that an unestablished meaning will be picked (see phases section in appendix for details on how to set the appropriate parameters). For example, the experimenter may want 50 % of topics to be unestablished and 50 % to be established. This is a crucial functionality, as if there are far more established meanings than unestablished one, and all meanings are equally likely to be seen, then it will take a long time for unestablished meanings to become established so that the experiment can progress.

### Client side interface

The server must be running when the client is launched. Upon launch, the client immediately starts a connection to the server. At first, participants will see a welcome screen that contains instructions, ideally detailing the structure of the experiment and explaining how to use the Leap Motion device, though we also recommend a live demonstration. The auditory feedback is available during the introduction screen so that the participant can practice and become familiar with the device.

In the communication game example, once there are two participants both of whom confirm they have read and understood the instructions by clicking an onscreen button, the experiment starts. The participants can then take turns in being the speaker and the hearer, producing and recognizing signals, respectively. However, whose turn it is to be speaker can also be assigned randomly, or dependent on other events in the experiment.

Participants may receive feedback about their communicative success between the turns, as well as information on the meaning that was intended by the speaker, and the meaning chosen by the hearer.

The experiment ends when participants have either successfully communicated all meanings, or the experimenter manually ends the session (see “Server side interface”), at which point participants are shown a message telling them the session is over and giving the appropriate instructions, e.g., leave the experiment booth.

### Client screens

Screens seen by the participants are designed to be as intuitive, fail-safe and user friendly as possible, even if participants have not read the instructions. All screens are displayed full screen without any window decorations such as minimize or exit buttons.

#### Simple text screens

Simple text screens can be added at the beginning of the experiment, or once before each experimental phase, if needs be.

#### Signal creation screen

On a signal creation screen, typically a participant will see a meaning for which they must create or reproduce a signal. This screen features the image, and a “Record” button, which can be pressed to start recording a signal. This turns into a “Stop” button, which can be pressed to stop the recording when the participant is done creating their signal. After the participant has created a signal, the button turns into a “Rerecord” button for if the participant is unhappy with their first recording. Participants can also play back a signal they have just created by pressing “Play”. When participants are happy with their signal they can then press “Submit”.

There is also a volume dial, so participants can readjust the volume should it be at an uncomfortable level. This has no bearing on the experimental data being logged, since that data consists of Leap Motion frames (hand position), not yet converted into audio.

#### Signal recognition screen

In communication game experiments, communicative success is measured by participants’ ability to correctly identify the meaning referred to by the signal of their partner. Signal recognition screens are also helpful in individual signal creation experiments. If the participant knows that they will be tested on their own signals, this creates an incentive for them to create signals that are distinct from one another. In this situation, if a participant is very bad at recognizing their own signals (at chance level) then this may be an indication that the participant is not taking the experiment seriously.

The signal recognition phase screen features one or two lines of instructions. Typically “Choose the image you think the signal refers to”, or similar. There is a “Play” button, which the participant can press to hear the signal to be recognized. There is also a set of possible meanings, which includes the target meaning. This set of images can have any number of elements, and can be chosen from the whole image collection, or from a finite subset of it. The size of this set can be modified. Finally, there is a “Submit” button to send the chosen meaning to the server.

This screen has certain restrictions to ensure valid responses. The participant is unable to press the submission button without listening to the signal at least once, preventing participants from guessing at random without knowledge of the signal. The participant must select an image before pressing submit, and only one meaning can be chosen before submission. Other dependencies are possible.

**Fig. 3:**
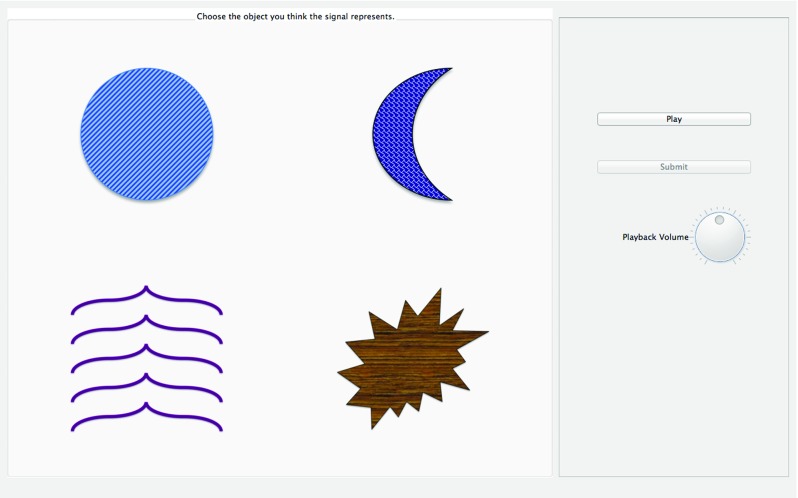
Signal recognition screen

The volume dial is also present on this screen (see Fig. 3), but does not need to be.

#### Transition screens

**Fig. 4:**
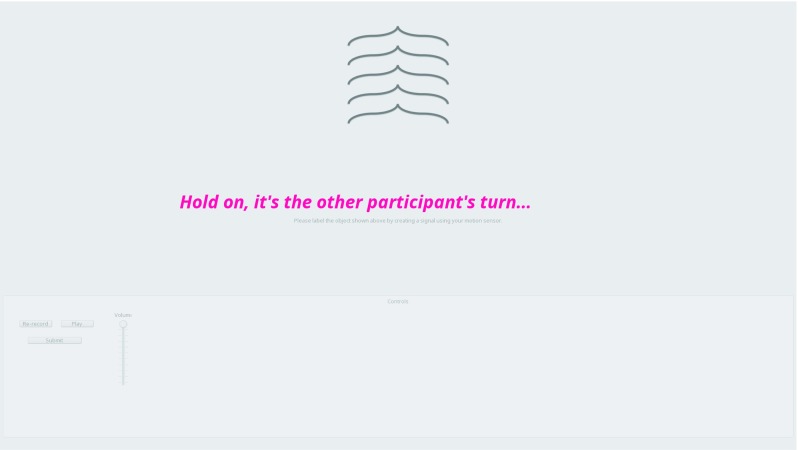
The wait dialogue after signal recognition

When a participant is waiting for the other participant to finish a task (such as creating a signal or recognizing one), they are shown filler screens (see Fig. 4). Since in the communication game participants take turns, it is important to make sure they are aware when it is not their turn, and that they are not allowed to interact with anything until it is. This “screen” is technically a semi-transparent modal dialogue that covers the current window. These screens are particularly useful during issues in networking where message passing might be delayed, and the participants tend to be confused unless they see explicit instructions to wait for the other participant.

### Server side interface

**Fig. 5:**
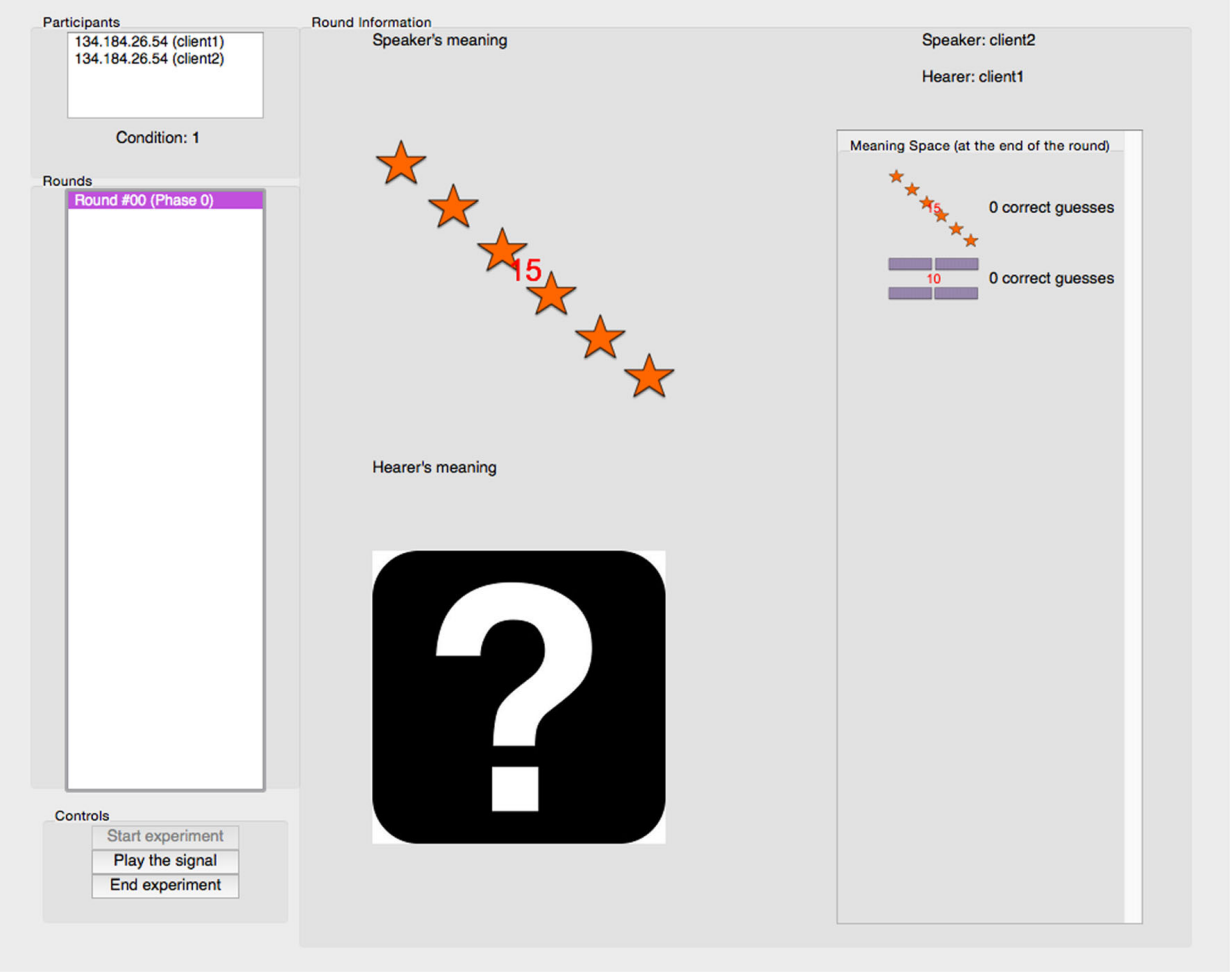
The server side interface as seen by the experimenter. The *red numbers* that can be seen on the meanings are only seen on the server side, and are the file names of the meanings

On the server side interface, each client connection is listed along with the address and unique ID of the participant (see Fig. 5). Once participants have indicated that they are ready on the client side, a new session starts.

The interface displays a list of rounds played in the experiment in real time, and clicking on each round displays a panel containing details such as the speaker’s intended meaning, hearer’s guess and speaker’s signal. It is also possible to playback the signal. Both the round list and the detail panel are updated live during the experiment.

It is possible to end the experiment at any moment using the provided *End Experiment* button. The server updates the log file at the end of every round, so ending the experiment early has no bearing on the recording of the data up until that point.

### Data Output

The data output is based on a single log file generated by the server. This file is updated at the end of each round. So if a session is aborted half way through for whatever reason, a full log of the session (except the unfinished, last round) is saved.

Log files are created that contain signal data and another containing question data. These objects, can be exported in one of the many formats, including comma separated values (csv). See Tables 1 and 2 for the columns available in the data frames.

**Table 1:** Columns of the response DataFrame, as returned by toPandas _p_2p() (see Appendix 1)

Response column	Description
round	Number of exchange, e.g., first exchange in the experiment is round 0.
phase	Number of phase.
client	client _*i*_ d of the signal creator.
image	What meaning was the signal produced for.
data_index	The index of this data point in the trajectory.
x	Hand position on the x-axis i.e., from left to right in millimeters
y	Hand position on the y-axis i.e., from down to up in millimeters
z	Hand position on the z-axis i.e., from front to back in millimeters
frequency	Frequency of the signal in Hertz. This is useful if you do not have a linear mapping between hand position and your auditory output.
mel	Frequency of the signal in Mel Scale.
amplitude	Volume of the signal in range [0,1]. This is useful if you do not have a linear mapping between hand position and your auditory output.

Other possible signal variables can be extracted from this output. For example, knowing the hand-position at each data frame within a signal will allow the experimenter to measure how much movement is in a signal or the mean pitch of a signal.^2^ Each frame is given an integer index starting from 0, so that the duration of each signal (i.e., the number of data frames) is simply the largest data_index value it is associated with.

### Online playback experiments

While it is true that it is more difficult to be iconic with continuous auditory signals than it is with graphical signals, iconicity can still exist, and be measured, in signals produced using the Leap Motion framework. In experimental semiotics, one way to measure iconicity is to let naïve participants see or listen to signals and have them pair them with an array of possible meanings (e.g., Garrod et al., 2007). If participants can pair signals with their intended meanings without any knowledge of how they were established, then those signals can be said to be iconic. For the most part, these playback experiments have been conducted online to allow for a massive number of participants, which increases statistical power. In order to make it easy to integrate the signals produced using the Leap Motion paradigm with such online experiments, we have built an interface that allows the experimenter to convert the log files to .wav files (see Appendix 1A.10). It is a small GUI application that allows the user to select a log file, and export all or some of the signals as wave audio files, allowing the experimenter to only select, for example, signals from a specific phase if they would like to compare iconicity of signals produced at the beginning of the experiment to signals produced at the end of the experiment. The application also allows the user to modify the playback rate (see Appendix 1A.4).

**Table 2:** Columns of the question DataFrame, as returned by toPandas_p2p() (see Appendix 1A.9)

Question Column	Description
round	Number of exchange, e.g., first exchange in the experiment is round 0.
phase	Number of phase.
client	client_id of the signal recognizer.
image0, image1, image2, etc.	Which meanings were in the set being selected from.
answer	The meaning that the signal was produced for.
given_answer	The meaning selected by the participant.
success	Are the answer and given_answer the same

## Limitations and further development

The primary limitation of this paradigm is its reliance on Leap Motion devices. Although the device itself is quite cheap (around $90 at time of writing), we cannot expect it to be widely available in people’s homes. This limits the applicability of the paradigm to laboratory settings where the hardware can be provided. Online experiments (which are becoming more and more prevalent) will not be feasible.

It is possible to use other, possibly native sensors (such as motion sensors of gaming consoles). However, that would require a major rewrite of the theremin component (see Appendix “A.1 Requirements”), but the component itself is quite small. Conceptually, the only change required is to make sure the callback method that receives data frames from Leap Motion receives the new data frames instead (see Appendix 1A.11 for details). Previous work has used infrared gaming sensors (such as the Xbox Kinect) to measure structure and conventionalization in gestural signals (Namboodiripad et al. 2016). The Leap Motion would be less suited to such work, as it has only one vantage point and doesn’t manage well when hands overlap.

The current framework implementation is limited in having exactly one exchange at a time. While this is often desirable, parallel interactions themselves can also be targets of research. This would require a major redesign of the current codebase to accommodate multiple interactions taking place at once. The main challenge would be to handle the parallel execution, and the increased complexity of tracking which meanings are established among which pairs. Moreover, each server instance can only handle a single session, but one can run as many server instances on a machine as possible, as long as each one listens at a different port.

The framework can accommodate more than two participants in a session, and its basic infrastructure can already initiate sessions with more than two participants. However, this has never been meaningfully tested since no such experiments have been implemented to date, and would require some additional customization to phases, e.g., in peer selection.

This framework is not necessarily tied to auditory signals mapping onto visual meanings: one can just as easily modify the framework to use visual representations of hand trajectories to serve as signals, and/or label sounds instead of images that serve as meanings. However, this would require extending the existing meaning- and signal-related classes and the UI to accommodate data from the new modality. The rest of the framework should work as outlined in this article. One restriction is that the meanings should already be present on all the computers participating in the session, so dynamically generated meanings are not supported by this framework. They need to be static and present from the onset.

Development within the Leap Motion framework has generally been progressing on an as-needed basis, and this is likely to continue to be the case. However, functionality for iterated learning experiments is a likely next step. The only significant change necessary is support for using the output of one session as the initial repertoire to reproduce in the next one, enabling the experiment to mimic generations of learners.

## Conclusions

We have developed a new signal space paradigm for conducting artificial language learning experiments investigating the emergence of combinatorial structure. This paradigm has improved on previous paradigms, as it allows for manipulation of the availability of iconicity, generates data that is easy to analyze, and is very flexible in terms of the size and shape of the signal space and the nature of the feedback. Ongoing work by the authors is utilizing the signal space flexibility to investigate the effects of the physical aspects of a signaling space in order to understand modality effects, something that was difficult using previous paradigms. Other uses of the paradigm include comparing visual signals to auditory ones, or replicating previous studies in experimental semiotics using auditory signals.

## Figures and Tables

**Fig. 6:**
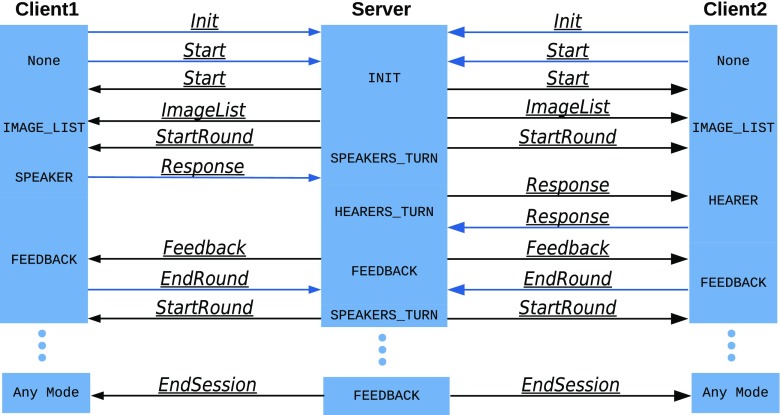
The message flow of a typical session. The Message suffixes in the class names are omitted here for brevity, so the Init class is actually called InitMessage

**Fig. 7:**
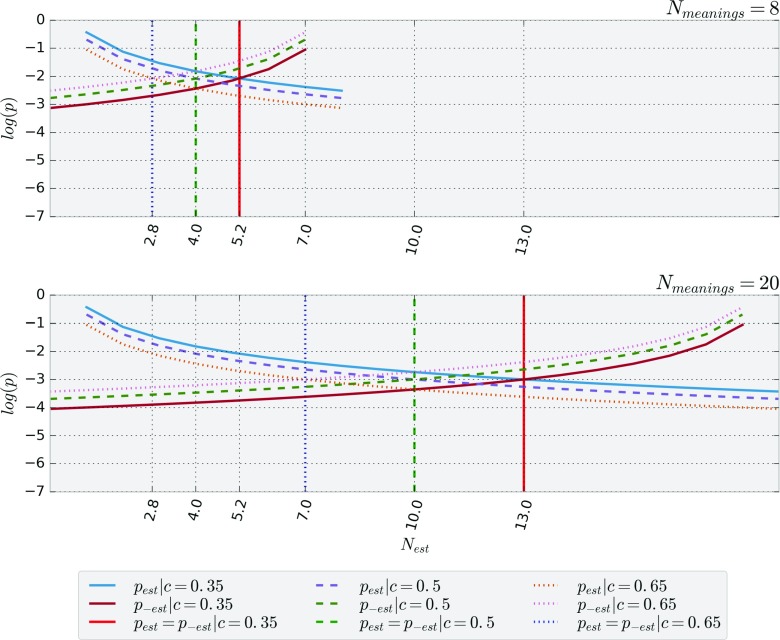
Plot of *l* *o* *g*(*p*_*est*_) and *l* *o* *g*(*p*_-*est*_) for two different levels of *c*, and different number of meanings *N*_*meanings*_. The x-axis is the number of established meanings

## Appendix A: Implementation

### A.1 Requirements

The framework itself is a package named leaparticulator.

The framework is designed for Python version 2.6 and higher, on Windows (7 and higher), Linux (any flavor, as long as you can get the libraries) or Mac OS/X (10.6 and higher). Python 3.x is not supported simply because several libraries the framework depends on are not ported to Python 3 yet; most critically twisted.

For some more isolated functionality of the framework, some dependencies may not be required. A version of Leap libraries for Windows, Linux and OS/X are already provided in the framework along with code that decides which one to use, but the Leap Motion driver needs to be installed separately.

For the analysis, a computer with multiple cores and at least 6 GB of memory is recommended. The scripts will spawn the right number of tasks based on the configurations it detects, and will use a single task if there is only one core. Memory usage is dependent on the data being processed, and care should be taken since the spawned tasks might fail if they run out of memory, possibly disrupting the whole batch of tasks and even the interpreter.

### A.2 Packages

The framework itself is a package named leaparticulator. The most important packages in the framework are:

- leaparticulator.browser: A tool to browse and visualize experimental data files.
- leaparticulator.data: Contains modules for representing and manipulating experimental data, including classes for representing Leap frames, meanings, trajectories of frames, and Hidden Markov Models, as well as functions for importing/exporting data files.
- leaparticulator.drivers: Contains Leap Motion libraries for various operating systems.
- leaparticulator.notebooks: Contains IPython notebooks and scripts used for data preprocessing, analysis and visualization, as well as other bits of code that don’t fit in anywhere else.
- leaparticulator.p2p: Contains the communication game implementation.
- leaparticulator.test: Contains tests for various classes and behaviors, as well as test data.
- leaparticulator.theremin: Contains the theremin implementations.
- leaparticulator.trajectory_recorder: Contains a tool to export trajectories in experimental log files as wave audio files.

The Python dependencies are the following, along with their roles (note that the libraries themselves might have non-Python dependencies, such as PyQt4 depending on Qt4) (Table 3):

**Table 3:** Dependencies of the framework

Dependency	Function
PyAudio	For audio I/O.
PyQt4	For the graphical user interfaces.
twisted	For asynchronous I/O and networking.
qt4reactor	To integrate QT and : event loops.
IPython	Mostly for data processing and visualization scripts.
pandas	Frequently used in data processing scripts.
matplotlib	Used for data visualization.
scipy	Used for its implementations of scientific functions such as gamma.
jsonpickle	Used to serialize and deserialize data.
ghmm	Used for training HMMs.

The various parameters and paths used in the framework are defined in a separate Python module named leaparticulator.constants, and are thus easily customizable. Any mention of *constant*s in the following sections is a shorthand for “member of module leaparticulator.constants”.

Finally, all instruction text on experimental screens are parsed from plain text files residing in a resources folder, which is specified by the constant P2P_RES_DIR.

### A.3 Structure of experiments

Message classes reside in the module leaparticulator.p2p.messaging, and each are initialized using the data required for the function of the message. When a message arrives, the recipient server or client reacts based on the *type* of message.

Both the server and the client objects keep track of their current state using their member variable factory.mode.^3^ Possible values are defined in the constants module (see Table 4).

**Table 4:** Server and client mode constants

Client Mode	Indicates	Server Mode	Indicates
INIT	Initialization.	INIT	Initialization.
SPEAKER	Client is speaker.	SPEAKERS_TURN	Signal creation
HEARER	Client is hearer.	HEARERS_TURN	Signal recognition
FEEDBACK	Client is receiving feedback.	FEEDBACK	Clients are receiving feedback.

For the client, most changes to the mode occur in the client’s incoming data callback method LeapP2PClient.lineReceived(self, line), which is called for each line received. This method checks the current mode, ensures the message received was of the expected type given the current mode, takes the necessary action, and finally changes the mode to its next state if necessary. The default flow is outlined in Fig. 6.

There are two exceptions where mode is set outside of this method on the client; speak(self) and hear(self, image) methods set the FEEDBACK mode, preparing the client to receive feedback. These exceptions are due to these mode changes not being triggered by incoming messages to the client (which lineReceived() is a callback for), but by outgoing messages triggered by the UI. The server uses its lineReceived() method the same way.

The specific server and client implementations can override the method LeapP2PServer.lineReceived(self, line) to customize the experiment’s structure and flow. It is important to keep to the same pattern of type checking to ensure the system is working as intended, and not out of contingencies. Note that some functionality is compartmentalized into methods of their own, either to reduce clutter, or to ensure they are callable from outside the lineReceived() method (such as via the UI).

If the server or client needs to call the UI at any time, for instance to show a screen, it can find a reference to the current UI using the attribute factory.ui. Both the server and client have specialized UI classes (under leaparticulator.p2p.ui) which offer methods for common tasks such as getting a reference for the currently active window, showing a wait dialogue, or displaying the signal creation screen. The class itself is not a part of UI, but rather a wrapper that creates windows and widgets by reading specifications from .ui files created in QT Designer. Both the root folder to search for .ui files and the names of specific files for each screen are specified in the constants module (constants named QT_DIR and *_WIN, respectively, where the wildcard stands in for a screen name).

Note that both the client and the server need to have access to the file-based resources (such as .ui files or images) at identical relative paths within the project folder. In other words, the “./img”, “./res” and “./qt_generated” folders need to be synced across the server and the client for everything to work as expected.

### A.4 Signals

#### A.4.1 Transformation of Signals

We used the following equations to generate tones from the x- and y-coordinates of the hand being tracked. Note the amplitude is a logarithmic function of the (absolute) y-coordinate, whereas the frequency is an exponential function of the x-coordinate. The amplitude is constrained to the interval [0,1], with values falling outside the range corrected to the nearest boundary.

$$amplitude = 1.1 - {\log{|y|} \over \log{250}} $$

$$frequency = 110 \times 3^{(|x + 200|) \over 200} $$

We could further use the same equations to map coordinates to pitch or amplitude for our analysis, in order to be able to have both the features of the auditory feedback and hand position as dependent variables.

#### A.4.2 Manipulation of duration

Momentary frame rate of the Leap Motion vary with CPU load, which is particularly problematic for tasks that produce a replay dependent on accurate temporal information of hand position, as in our framework. To solve this, we developed an alternative listener that polls the device at preset intervals (set at constants.THEREMIN_RATE) and therefore samples at uniform intervals. This alternative implementation also helps constrain the maximum duration of the signals, which can be useful in situations where a pressure for shorter signals is required (as in e.g., Little et al., 2016b). Using the ConstantRateTheremin implementation ensures that our sampling is uniform, and that the playback rate is identical to the recording rate. The constants used specifically for this setup are MAX_SIGNAL_DURATION and THEREMIN_RATE. The former is the maximum signal duration in seconds, and the latter is the rate (with a unit of *s* *e* *c* *o* *n* *d* ^-1^) at which the audio will be updated based on the position of the hands. For instance, a theremin with a rate of 100 can change pitch one hundreds times every second (the recommended default). Note that very high rates can be CPU-intensive. It is also important to note that you need to pass default_rate=THEREMIN_RATE to your ThereminPlayback constructor to ensure the playback is consistent with the recording.

With this boilerplate in place, it is relatively simple to put together a limited duration constraint. There already exists code to stop a recording, as LeapP2PClientUI.stop_recording(). This method is normally bound to the click events of “Stop recording” buttons, but it can also be used as a generic way to stop recording a signal. To limit the duration, all one needs to do is to set MAX_SIGNAL_DURATION to a positive value, and then twisted will call stop_recording() after waiting MAX_SIGNAL_DURATION seconds. The appropriate Theremin and ThereminPlayer instances are set up under the hood. Setting MAX_SIGNAL_DURATION to a non-positive value such as 0 turns off the duration constraint.

Implementing a simple progress bar (as seen in Fig. 2), so that participants can see signal time elapsing is a useful addition. Assuming there already is a QProgressBar widget on your window, and that it is referenced by self.progressbar of your UI object, it is possible to update the progress bar in intervals of about .1 seconds as follows:

### A.5 Phases

For experiments where the meaning grows or changes from phase to phase, the index of the last item in the currentexpansion state is kept in an integer variable called image_pointer, on both the client and server. Each expansion changes the meaning space (or possibly the signal space if the experimental design demands this) and therefore constitutes a new phase. The meaning space is kept in a list called images on both the server and client factory objects.

The entry point for meaning space expansion is the expandMeaningSpace() method of the server object. This method both checks whether or not it is necessary to perform an expansion (i.e., if all meanings in this phase are established), and then performs one if necessary. Finally, it sets LeapP2PServerFactory.end_experiment to True if an expansion is warranted yet impossible (i.e., if all possible meanings are established). The number of successful communications needed before a meaning is “established” can be set using the LEARNING_THRESHOLD in constants. The extent of the expansion can be modulated by the constant MEANING_INCREMENT, which specifies the increment that will be added to image_pointer at each expansion.

At each round, the method choose_speaker_and_topic() of the server is called to determine the speaker, as well as choose a topic from the meaning space. Currently, it is set to choose topics based on how well established the topic-signal pairing is, as detailed below. To customize this behavior beyond what current parameters allow, such as constraining the number of times a topic can be chosen, or just presenting topics randomly, the choose_speaker_and_topic() method can be re-implemented.

The NOVELTY_COEFFICIENT is the probability of picking an unestablished meaning for a round. For example, if this value is 0.5, then 50 % of meanings communicated will have already been established at the point they are communicated (if there are any established meanings, otherwise it defaults to only unestablished meanings), and 50 % will be unestablished.

For *N*_*e**s**t*_ established meanings in a meaning space of *N*_*m**e**a**n**i**n**g**s*_ items, and a NOVELTY_COEFFICIENT *c*, the probabilities for picking each item established meanings can be calculated as follows:

1 $$\begin{array}{@{}rcl@{}} p_{est} &=& (1 - c) N_{est} - 1\\ p_{-est} &=& c N_{est} - 1\\ \end{array} $$

To find the number of established meanings *N*_*e**s**t*_ where *p*_*e**s**t*_ = *p*_-*e**s**t*_ (after which unestablished items get increasingly frequent), it is sufficient to solve:

2 $$\begin{array}{@{}rcl@{}} \frac{1-c}{N_{est}} &=& \frac{c}{N_{meanings} - N_{est}}\\ N_{est} &=& N_{meanings} (1 - c)\\ \end{array} $$

The initial pressure towards choosing established meanings is being replaced by one towards choosing unestablished meanings in the end (see Fig. 7). Whenever there is a difference between the number of established and unestablished meanings, the bias will favor the smaller group. Initially, this helps the participants keep practicing the few established meanings that they will hopefully build on. Towards the end of a phase, it helps participants focus on unestablished meanings in a mostly-established meaning space.

We recommend a NOVELTY_COEFFICENTS above 0.5 (the default is 0.55), reducing the minimum number of meanings to establish before the pressure towards unestablished meanings prevails. Fine tuning this value is particularly important for larger meaning spaces, which can quickly get either too repetitive or too novel for the participants.

### A.6 Client Side

The client interface is started by running leaparticulator.p2p.server with the following command (assuming the current working directory is in PYTHONPATH):

The parameter condition may not be useful in experiments without conditions, but a value is still required to ensure the server and client agree, and may be useful if new conditions are implemented in the future. The client keyword tells the module we are trying to spawn a client and not a server, and the client_id is used as a unique ID for this client on the server, for example, a participant number. Providing non-unique IDs within an experiment with more than one participant causes an exception to be thrown during the connection attempt.

It is also possible to provide the server address directly from the command line, overriding the server details in the constants file, although this is optional.

#### A.6.1 Text Screens

Most experiments start with a simple text screen explaining the experiment, or giving other relevant information. Such a screen can be shown by invoking LeapP2PClientUI.first_screen(), and the final screen (e.g., saying thank you for completing the experiment or giving further instructions) can be shown using final_screen() of the same class. By default there are only two information screens, an initial one containing instructions and another one concluding the experiment, both of which are shown exactly once.

Text for these screens are kept in the files “res/p2p/*_screen.txt”, named after the name of the screen. and read in when the screen is being constructed. Images can be added to this screen by editing the file indicated in the constant FIRST_WIN.

Any change that is more involved than that requires some (very simple) coding. If the experiment requires multiple text screens (e.g., to display the new meaning space at each phase change), one can easily create and customize clones of this screen and call them at appropriate times (e.g., between different phases). The rest of the experiment should not be affected. The right place to add new screens is the lineReceived() method of the client, like most changes to the experiment’s flow.

If these screens are not remarkably different in structure to simple text screens, we suggest implementing a general information window that displays the right information based on an argument provided. For instance, if we wanted to make the first and last screens more general, and add an info screen before each phase, we would need to do the following:

1. Remove the previous info screens.
2. Create the UI file for the generic window. This window should include the text containers to be populated later.
3. Create the relevant text resources in the resources folder.
4. Create constants to mark the different modes.
5. Create a method that will populate the window with the relevant information.
6. Ensure the new screen will be followed by the correct screen when closed.
7. Call the screen from the previous step as needed.

As an alternative to using textual content that requires formatting, it is also possible to provide or even build the window contents as an image file, and using a simple screen with an image container for the same purpose we use the text containers in the examples above. For instance, it is possible to add a montage of the meaning space at the beginning of a phase by simply creating the image using external tools (such as Imagemagick’s montage command), and using it to populate the screen. Note that this strategy may not work well with displays of different sizes due to image scaling.

#### A.6.2 Signal creation screen

The signal creation screens are pretty straightforward (see Fig. 2). The entry point for this screen is the method creation_screen(image=None) of LeapP2PClientUI. The argument image must be a subclass of the class AbstractMeaning from module leaparticulator.data.meaning, and indicates the meaning that the participant will generate a signal for. The .ui file used to build the window is stored in the constant CREATION_WIN.

#### A.6.3 Signal recognition screen

For signal recognition screens (see Fig. 3), the maximum number of options available per question can be changed using the constant N_OPTIONS. One can also customize the interface itself by editing the file indicated in constant *TEST_WIN*. The testing window must always have exactly N_OPTIONS QPushButton objects, and they should be named btnImageN where N is the option number.

Also, if N_OPTIONS is greater than 4, some functions under the package leaparticulator.data (most notably those producing Pandas frames from log files, see “Data Output”) would need modification to extract the new columns from the log file as required by the extra options. Once these are in place, the rest will be handled under the hood.

The main point of entry for this screen is the method LeapP2PClientUI.test_screen(). The selection of the options that are not the target meaning is at random by default. One can customize it at LeapP2PServer.lineReceived().

#### A.6.4 Transition screens

Can be shown using the show_wait() methods of UI objects. Showing any other screen automatically disables this dialogue, as well as calling wait_over(). The UI file can be changed using the constant WAIT_WIN.

### A.7 Server side

The server side interface must be launched before the client side. It is started by running the module leaparticulator.p2p.server with the following command (assuming the current working directory is in PYTHONPATH):

The uid command line argument dictates the name of the log file. The researchers can use it to implement whatever scheme they use to label experimental runs. If omitted, the unique ID is generated from the timestamp at the moment of execution.

Upon launch, the interface immediately fires up a new server instance listening on the port specified in the constants module. Each new connection is listed along with the address and unique ID of the participant, and upon confirmation from both parties that they are ready on the client side, a new session starts.

The interface is read in from the file indicated by the constant SERVER_WIN.

### A.8 Meanings

Images that will be used as meanings can be placed in the ”img” folder (or any other folder as specified in the constant MEANING_DIR_P2P) and can be selected specifically or at random depending on the experimental design. This logic resides in the constructor for LeapP2PServer, and the default behavior is to randomize the meanings.

When a session starts, the server checks the images it is able to find in the meanings directory based on a given file name pattern stored in the constant P2P_IMAGE_MASK, and calls the FromFile() class method of leaparticulator.data.P2PMeaning for each filename found. One can subclass the class AbstractMeaning of the same module to create new meaning classes.

It is also possible to pass the parameter max_images to the server factory constructor to limit the number of meanings.

For experiments that have custom requirements for phases (see “Phases”), it is sufficient to override the method expandMeaningSpace() of the class LeapP2PServer to implement behaviors that go beyond simple expansion, such as replacement of the whole meaning space.

### A.9 Data output

The name of the log file is dictated by the LeapP2PServerFactory.uid attribute (see “Server side interface”). If one is not provided, the current timestamp is used instead. The log file is output to the LOG_DIR subdirectory of the ROOT_DIR, which is expected to be the root folder of the project.

The file consists of several JSON^4^ objects, one per line. For an experiment of *N* rounds, the log file would consist of *N*+2 lines. The first two lines describe the participants and the image list (i.e., the meaning space in its maximally expanded form), and every line after that contains a LeapP2PRoundSummary object serialized into JSON, one for every round.

An overview of the attributes of a LeapP2PRoundSummary object is below (Table 5):

**Table 5:** Attributes of a LeapP2PRoundSummary object

Attribute	Description
speaker	Speaker’s client_id
hearer	Hearer’s client_id
signal	Raw data from sensor
image	Speaker’s target image
guess	Hearer’s guess
success	True *iff* image equals guess
options	Options in the recognition screen excluding target
success_counts	dict from meanings to consecutive success counts
image pointer	Index of the last element in the meaning space

Note that round number is not an explicit part of the logged data. This information is still present: the summary at line *n* is always for round *n*-2.

At the analysis stage, the log file needs to be read into a convenient data structure. Our framework offers a way of doing this using the function leaparticulator.data.functions.toPandas_p2p. This method takes a log file path as a parameter, and returns a tuple of two pandas.DataFrame objects: one containing response data (i.e., data regarding the signal creation task), and another containing question data^5^ (i.e., data regarding the signal recognition task). These objects, in turn, can be exported in one of the many formats pandas.DataFrame supports, such as CSV. See Tables 1 and 2 for the columns of the DataFrame.

### A.10 Signal recorder

The module leaparticulator.trajectory_recor der.recorder_p2p handles this conversion of signals to .wavs. The module can be run directly from the command line, and the argument “constantrate” should be passed if the constant rate theremin implementation is needed. The user interface is quite straightforward, and consists of browsing for files, choosing some, browsing for the target directory, and hitting a button to export them.

### A.11 Using other sensors

Using other sensors to feed data into this framework should be quite simple. In particular, let’s consider plugging in another sensor into the theremin. All the work required is concentrated to the actual theremin module. The method Theremin.on_frame(frame) handles everything from dispatching the frames on to the ThereminPlayer instance to adding them to the appropriate buffer if there is an ongoing recording. It might be best to copy its behavior as closely as possible while integrating the data stream from the new sensor. The only thing left to do is extending ThereminPlayer to convert from whatever features your sensor provides to an audio signal. How exactly to realize this conversion is, of course, up to the researcher.

## Footnotes

1
^1^ In Verhoef et al. (2015b) the researchers analyzed some of the data as it was being produced in real time, which was especially useful because some of the data was collected as part of a public exhibition. Accordingly, it was good for participants or visitors to be able to see the data being produced and analyzed in real time.

2 Note that the data frames produced by Leap Motion contain considerably more information than included here, including timestamps for each frame; see the Leap Motion documentation for a comprehensive list of the types of information these frames contain.
